# A review of physiological functions of orexin: From instinctive responses to subjective cognition

**DOI:** 10.1097/MD.0000000000034206

**Published:** 2023-06-30

**Authors:** LiBo Xia, Hai Yan Liu, Bi Yan Wang, Hai Ning Lin, Meng Chen Wang, Ji-Xiang Ren

**Affiliations:** a Department of Encephalopathy, Jilin Provincial Hospital of Chinese Medicine, Changchun, China; b Department of Medical Section, Changchun Second Hospital, Changchun, China.

**Keywords:** addiction, anxiety, depression, feeding, orexin, wakefulness

## Abstract

Orexin, also known as hypocretin, is an excitatory neuropeptide secreted by the hypothalamus. Orexin is divided into orexin-A (OXA) and orexin-B (OXB), which are derived from a common precursor secreted by hypothalamic neurons. Orexin acts on orexin receptor-1 (OX1R) and orexin receptor-2 (OX2R). Orexin neurons, as well as receptors, are widely distributed in various regions of the brain as well as in the peripheral system and have a wider range of functions. This paper reviews the latest research results of orexin in the aspects of food intake, sleep, addiction, depression and anxiety. Because orexin has certain physiological functions in many systems, we further explored the possibility of orexin as a new target for the treatment of bulimia, anorexia nervosa, insomnia, lethargy, anxiety and depression. It is precisely because orexin has physiological functions in multiple systems that orexin, as a new target for the treatment of the above diseases, has potential contradictions. For example, it promotes the function of 1 system and may inhibit the function of another system. How to study a new drug, which can not only treat the diseases of this system, but also do not affect other system functions, is what we need to focus on.

## 1. Introduction

Orexin/ Hypocretin is an excitatory neuropeptide that was first discovered in 1998 by 2 independent research groups through different research modalities. Sakurai used chromatography to discover a novel neuropeptide, which was named “orexin” because of its ability to enhance food intake. Almost at the same time, another group also discovered this peptide using molecular biology techniques, since this peptide is secreted by the hypothalamus hence the name “hypocretin.” To avoid confusion, the term orexin is used in the following accounts.

Orexin is divided into orexin-A (OXA) and orexin-B (OXB), which are derived from a common precursor secreted by hypothalamic neurons. The structure of OXA is highly conserved among mammals, suggesting an important role^[1]^; but the OXB amino acid sequence has some differences among different species.^[2]^

It is estimated that 3000 to 6700 neurons express orexin in the rat brain, whereas approximately 50000 to 80000 neurons express orexin in the human brain.^[3]^ These neurons project widely to the central nervous system. Most project in regions controlling wakefulness, including the locus coeruleus,^[4]^ dorsal raphe nuclear region.^[5]^ Of course also project to other regions, including the basal forebrain (BF), olfactory region, bed nucleus of the stria terminalis,^[6,7]^ indicating that orexin has additional functions beyond controlling wakefulness.

There are 2 orexin receptors known, orexin receptor-1 (OX1R) and orexin receptor-2 (OX2R). OX2R is present in all vertebrates, whereas OX1R is present only in mammals, so OX1R may have been a product of biological evolution.^[3]^ OXA has equal affinity for both receptors, whereas OXB has 10-fold greater affinity for OX2R than OX1R. OX1R is mainly distributed in areas that control feeding, learning and memory, and reward,^[8]^ whereas OX2R is mainly found in areas that control arousal.^[9]^ It can be speculated from the distribution of orexin neurons and receptors that the orexin system has a wider range of functions.

## 2. Regulation of feeding and energy balance

Orexin achieves a homeostatic state of energy by regulating feeding and voluntary movement to affect energy intake, storage, and expenditure. Orexin increases feeding desire and food seeking behavior to replenish energy when the body is energy deficient; Increasing the level of body movement and energy expenditure in times of energy excess.

In initial studies, it was found that the orexin system is implicated in the regulation of food intake^[10]^, and the lateral hypothalamus is a brain region involved in the regulation of food intake that is enriched with a large number of neural and orexin receptors that can produce orexin. Studies in rats have shown region specific damage to the hypothalamus that leads to alterations in food intake, or to overeating and obesity, or to a decrease in food intake, ultimately leading to death.^[11]^ Microinjection of OXA into the hypothalamus increases food intake.^[12]^ Compared with the control group, intraperitoneal injection of selective OX1R antagonist SB-334867 (10, 20, or 30 mg/kg) significantly reduced the incidence of overeating in mice (*P* < .01).^[13]^ Combined with the above localization evidence, and experimental observations in animals illustrate that the orexin system can contribute to animal feeding.

Rather than simply, indiscriminately, and restrictively increasing appetite, the orexin system is a food reward, a form of food seeking behavior that is tuned to energy demand, and the suitability of food is the primary factor driving this behavior. For example, in the absence of energy, the orexin system promotes the body to preferentially seek and eat foods rich in sugars and fats.^[14,15]^ It has been shown that when mice are approached to chocolate, orexin cells produce a significant calcium signal,^[16]^ suggesting that the consumption of a suitable food can increase the activity of orexin neurons to promote eating behavior.

Orexin system can promote dietary intake and may contribute to binge eating behavior. Accordingly, orexin receptor antagonists can suppress binge eating symptoms, and Piccoli et al^[17]^ found that an OX1Rantagonist selectively reduced binge eating of palatable foods without affecting normal food intake without inducing sleep. ACT-539313 is an OX1Rantagonist used for several indications, including binge eating disorder. For the first time, data have been published for this drug in humans (Compared with plcebo, *P* = .0190 < .05).^[18,19]^

Now that orexin receptor antagonists can suppress binge eating symptoms, activating the orexin system can treat anorexia; however, the role of the orexin system in anorexia nervosa is unknown. Two diametrically opposite results emerged in studies, with 1 finding altered orexin levels in the plasma of anorexia nervosa patients, while others did not.^[20]^ Some animal experiments have found that subcutaneous injection of OX2R selective agonist-YNT-18 in mice can significantly alleviate the anorexia caused by chemotherapy in mice; Compared with the control group, *P* < .0001.^[21]^ OX1R agonists have also achieved some efficacy in the treatment of anorexia nervosa, but the efficacy was not significant,^[22]^ suggesting that our application of dual receptor agonists may be a future research direction for the treatment of anorexia nervosa.

The orexin system is able to increase food intake, but also the level of body movement and energy expenditure, and resist the development of obesity. Orexin induced eating is inhibited by glucose, triglycerides, and amino acids. The orexin system regulates energy metabolism balance by monitoring physiological changes in glucose levels, which are stimulated to be activated when extracellular glucose concentrations are low below a threshold.^[23]^ Increased extracellular nonessential amino acids (a consequence of energy production from protein breakdown) also affect the activity of orexin neurons, depolarizing them.^[24]^ Both OX1R and OX2R have been observed in human adipose tissue, and orexin confers higher rates of lipolysis.^[25]^ Illustrates that the orexin system is a sensor of the metabolic and nutritional status of animals.

Orexin system can promote body movement to consume excess energy, and proper movement can also promote the secretion of OXA. At the same intensity of exercise, orexin deficient mice appeared to gain weight, whereas normal mice gained almost unchanged weight, indicating that orexin neuronal signaling is dually affected by diet and exercise in weight regulation.^[26]^ Physical exercise can increase plasma OXA levels, thereby activating the sympathetic nervous system and energy expenditure.^[27]^ Orexin system can regulate energy expenditure, increase autonomic activity and reduce sedentary time,^[28]^ and higher plasma OXA levels in humans with moderate exercise were found in obese and overweight subjects.^[29]^

Based on the above findings, the orexin system may be an important system for controlling feeding, regulating energy balance, and positively contributing to the promotion of physical health.

## 3. Maintenance of wakefulness

The orexin system plays a key role in maintaining the state of wakefulness and regulating the transition from the sleep state to the wakefulness state.^[30]^ Orexin receptor mutations were initially identified in dogs with narcolepsy; It was later observed that following knockout of the pre orexin gene in mice, the mice exhibited lethargy with episodic cataplexy symptoms.^[31]^ And further demonstrated that injection of orexin could improve the symptoms in narcolepsy animals^[32]^; Conversely, orexin receptor antagonists promote sleep and cataplexy in narcolepsy mice.^[33]^ It follows that orexin and its receptors play a critical role in maintaining the arousal state.

Neuroanatomical studies demonstrate widespread projections of orexin containing neurons to key brain regions involved in the regulation of the sleep wake cycle, maintaining humans in a wake-up state by stimulating wake promoting regions. Orexin activates the cerebral cortex, driving wakefulness, by stimulating BF cholinergic neurons.^[34]^ Injection of orexin to the BF stimulates wakefulness, reducing NREM sleep,^[11]^ because orexin neurons couple to BF cholinergic neurons, depolarizing them, a pathway that plays a key role in regulating wakefulness. Orexin neurons project densely onto locus coeruleus noradrenergic neurons, regulating their activity via orexin. Noradrenergic neurons have their specific firing pattern, with the highest firing frequency during wakefulness and a lower frequency during non-rapid eye movement sleep but not during rapid eye movement sleep,^[35]^ thus, orexin indirectly controls wakefulness by affecting ne neurons. In addition, orexin can also increase the firing rate of neurons and prolong wakefulness by stimulating other regions of the brain, such as the dorsal raphe nuclear region.^[12]^ All of these neuronal pathways demonstrate the complex role of the orexin system in the regulation of the arousal state, but the arousal system requires a large number of neuronal interconnections, as well as the interaction of various neurotransmitters, in which the mechanism of action of the orexin system is not well defined, and this will be the focus of future studies.

The answer is no whether orexin 2 receptors play the same role in maintaining wakefulness. Mice lacking the OX2R receptor have narcolepsy manifestations, whereas knockout OX1Rmice show only mild sleep disturbances^[36]^; however, the sleep wake cycle defect in the knockout dual recipient mice is more severe than in the knockout OX2R only mice.^[37]^ Thus, it could be shown that both OX1R and OX2R are essential for the maintenance of wakefulness, with the role of OX2R being more important.

Understanding the role of the orexin system in the sleep wake cycle may guide clinical development of drugs to treat sleep disorders. OX2R agonists may be useful in the treatment of narcolepsy. Conversely, antagonists of OX2R can be used to initiate and prolong sleep. A randomized, double-blind, placebo-controlled clinical study showed that the use of OX2R antagonists alone was sufficient to promote human sleep. OX2R antagonists increased the average time of REM and non-REM sleep, *P* < .05^[38]^ compared with the control group. However, the choice of OX1Rantagonist or OX2R antagonist, or dual receptor antagonist, for the treatment of insomnia still requires a large number of animal experiments and clinical trials to analyze the contrast.

## 4. Regulating emotions

### 4.1. Addiction

Addiction is a compulsive, persistent reward seeking behavior that may even reshape neuronal circuits. The role of the orexin system in reward seeking behavior underlies its role in addiction. In the preceding text it was described that in the absence of heat the orexin system promotes the body to preferentially seek out and eat foods rich in fat and sugar. This food seeking behavior is a reward seeking behavior; Binge eating disorder patients can be simply understood as addictive to food because these patients have an undruggable desire to eat.

Orexin activates reward pathways by upregulating addiction associated neurotransmitter and receptor activity. Addiction and reward behaviors are often accompanied by enhanced neurotransmitter transmission within the mesocorticolimbic system that facilitates the release of dopamine,^[39]^ which provides pleasure, activates reward pathways and thereby motivates repetitive activity, ultimately producing addiction. Dopamine levels are significantly increased in the brains of mice chronically consuming morphine and are gradually restored to normal levels by blocking OX1Rfunction.^[40]^

Drug addiction increases orexin neuron numbers. Chronic cocaine administration causes a significant increase in the number of orexin neurons.^[41]^ Adult rats, after chronic alcohol gavage, the orexin system is upregulated, expressing more orexin^[42]^; Zebrafish maternal alcohol consumption before fertilization significantly increased the number of orexin neurons by 18% (*P* < .01) compared with the control group, and increased the amount of alcohol consumption of offspring (*P* < .001), and the amount of alcohol consumption of offspring was positively correlated with the number of orexin neurons, which was not found in the control group.^[43]^ This suggests that addiction dominated by the orexin system may have a genetic effect. Increased numbers of orexin neurons are also induced after opioid abuse. Dissection of brain tissue lethal from heroin addiction found a half increase in the number of orexin neurons compared with controls.^[44]^ In addition chronic use of opioids such as morphine and fentanyl both result in increased numbers of orexin neurons.^[45,46]^

The increased number of orexin neurons is a big characteristic of addiction, so we can treat the addiction by inhibiting the secretion of orexin or by using antagonists. Found that mice with a knockout of the orexin gene had attenuated morphine dependence and that orexin antagonists blocked drug seeking behavior. OX1R selective antagonists can significantly reduce opioid use motivation and drug seeking behavior (*P* < .0001).^[47]^ Orexin system is associated not only with addiction but also with withdrawal syndromes. Found that the somatic symptoms of nicotine withdrawal were attenuated using mice with an OX1Rantagonist and orexin knockout mice (*P* < .01).^[48]^

Long term medication use or substance abuse can affect nutritional status and may lead to sleep disturbances. Therefore, targeted regulation of orexin system may be an effective strategy to reduce drug intake and promote food intake and sleep normalization. Because of the major physiological role of OX2R in the regulation of wakefulness, most modern research focuses on investigating the potential anti addiction effects of selective antagonists of OX1R, but the choice of single or dual receptor antagonists remains inconclusive.

### 4.2. Depression

Found lower orexin levels in animal models of depression and in patients with major depressive disorder than in normal healthy controls. The selective OX1Rantagonist was chosen to treat the animals and was unexpectedly found to cause depressive symptoms in control animals^[49]^; The expression of OX2R in their hypothalamus was found to be lower in mice with depressive symptoms.^[50]^ A study evaluating the level of OXA in cerebrospinal fluid found a significant decrease of OXA in cerebrospinal fluid of patients with major depressive disorder as well as some patients who attempted suicide due to major depression compared to healthy individuals (*P* < .01).^[51]^

Depression is a disease in which prolonged depressed mood is the primary manifestation, and the orexin system affects the organism state of wakefulness and can activate reward pathways, which holds promise as a new target for the treatment of depression.

### 4.3. Anxiety disorders

Increased orexin levels are associated with anxiety like behavior in animals. Orexin was found to induce panic, anxiety like behaviors in animal models.^[52]^ Conversely, in an animal panic model, OX1R antagonist treatment reduced panic like behavior and alleviated anxiety inducing symptoms.^[53]^ A study involving 56 drug-free adolescents diagnosed with any anxiety disorder other than specific phobias and 32 healthy controls found that the OXA level of anxiety patients was significantly higher than that of the control group (*P* < .001).^[54]^

**Figure 1.**
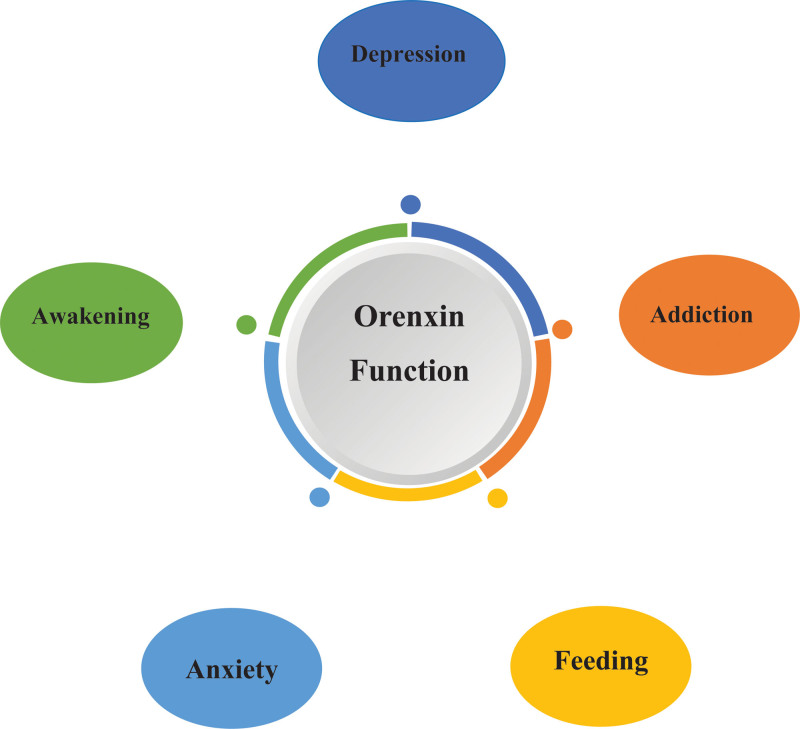
Orexin physiological function map.

Anxiety disorder is a disorder characterized by intense, excessive, and persistent worry or fear, accompanied by symptoms such as palpitations and difficulty sleeping. Orexin is an excitatory neuropeptide that keeps the organism awake and under stress, and it was found that orexin levels were higher in patients with anxiety disorder than in normal healthy people. Orexin receptor antagonists therefore hold promise as novel targets for the treatment of anxiety disorders (Fig. 1).

## 5. Summary and Outlook

1. Orexin plays a certain role in regulating food intake, maintaining arousal and regulating mood, but OX1R and OX2R have different physiological functions; So when designing drugs, should we choose single receptor or double receptor?
2. Orexin agonists can treat depression, anorexia and other diseases, but can cause insomnia. Orexin antagonists can be used to treat insomnia, alcohol and drug addiction, anxiety and other diseases, but it will reduce food intake and lead to malnutrition. How to balance these contradictions?
3. will long-term use of orexin agonists or antagonists lead to drug resistance and serious side effects?

The above 3 issues also need a large number of animal experiments and human experimental research summary data.

1. There is little literature on whether there are gender differences in orexin secretion, which may be a direction for future research.

## Author contributions

**Conceptualization:** LiBo Xia.

**Methodology:** Hai Yan Liu.

**Supervision:** Ji-Xiang Ren.

**Visualization:** Bi Yan Wang.

**Writing – original draft:** LiBo Xia.

**Writing – review & editing:** LiBo Xia, Ji-Xiang Ren, Hai Ning Lin, Meng Chen Wang.

## References

[1] Grady FS Boes AD Geerling JC. A century searching for the neurons necessary for wakefulness. Front Neurosci. 2022, 16: 930514. 35928009 10.3389/fnins.2022.930514 PMC9344068

[2] Wong KK Ng SY Lee LT. Orexins and their receptors from fish to mammals: a comparative approach. Gen Comp Endocrinol. 2011;171:124–30. 21216246 10.1016/j.ygcen.2011.01.001

[3] Soy S Sakurai T. Evolution of orexin neuropeptide system: structure and function. Front Neurosci. 2020;14:691. 32754010 10.3389/fnins.2020.00691 PMC7365868

[4] Soya S Takahashi TM McHugh TJ. Orexin modulates behavioral fear expression through the locus coeruleus. Nat Commun. 2017;8:1606. 29151577 10.1038/s41467-017-01782-z PMC5694764

[5] Yang C Zhang L Hao H. Serotonergic neurons in the dorsal raphe nucleus mediate the arousal-promoting effect of orexin during isoflurane anesthesia in male rats. Neuropeptides. 2019;75:25–33. 30935682 10.1016/j.npep.2019.03.004

[6] Lebow MA Chen A. Overshadowed by the amygdala: the bed nucleus of the stria terminalis emerges as key to psychiatric disorders. Mol Psychiatry. 2016;21:450–63. 26878891 10.1038/mp.2016.1 PMC4804181

[7] Kaminski T Smolinska N. Expression of orexin receptors in the pituitary. Vitam Horm. 2012;89:61–73. 22640608 10.1016/B978-0-12-394623-2.00004-4

[8] Pantazis CB James MH O’Connor S. Orexin-1 receptor signaling in ventral tegmental area mediates cue-driven demand for cocaine. Neuropsychopharmacology. 2022;47:741–51. 34635803 10.1038/s41386-021-01173-5 PMC8782853

[9] Liblau RS Vassalli A Seifinejad A. Hypocretin (orexin) biology and the pathophysiology of narcolepsy with cataplexy. Lancet Neurol. 2015;14:318–28. 25728441 10.1016/S1474-4422(14)70218-2

[10] Lee J Raycraft L Johnson AW. The dynamic regulation of appetitive behavior through lateral hypothalamic orexin and melanin concentrating hormone expressing cells. Physiol Behav. 2021;229:113234. 33130035 10.1016/j.physbeh.2020.113234 PMC8041470

[11] van Galen KA Ter Horst KW Serlie MJ. Serotonin, food intake, and obesity. Obes Rev. 2021;22:e13210. 33559362 10.1111/obr.13210 PMC8243944

[12] Michael NJ Elmquist JK. Coordination of metabolism, arousal, and reward by orexin/hypocretin neurons. J Clin Invest. 2020;130:4540–2. 32804153 10.1172/JCI140585 PMC7456245

[13] Alcaraz-Iborra M Carvajal F José Manuel L-C. Binge-like consumption of caloric and non-caloric palatable substances in ad libitum-fed C57BL/6J mice: pharmacological and molecular evidence of orexin involvement. Behav Brain Res. 2014;272:93–9. 24983661 10.1016/j.bbr.2014.06.049

[14] Muthmainah M Gogos A Sumithran P. Orexins (hypocretins): the intersection between homeostatic and hedonic feeding. J Neurochem. 2021;157:1473–94. 33608877 10.1111/jnc.15328

[15] La Marra M Caviglia G Perrella R. Using smartphones when eating increases caloric intake in young people: an overview of the literature. Front Psychol. 2020;11:587886. 33343462 10.3389/fpsyg.2020.587886 PMC7744612

[16] Mitchell CS Fisher SD Yeoh JW. A ventral striatal-orexin/hypocretin circuit modulates approach but not consumption of food. bioRxiv. 2020;14:603245.

[17] Piccoli L Micioni Di Bonaventura MV Cifani C. Role of orexin-1receptor mechanisms on compulsive food consumption in amodel of binge eating in female rats. Neuropsychopharmacology. 2012;37:1999–2011. 22569505 10.1038/npp.2012.48 PMC3398727

[18] Kaufmann P Ort M Golor G. Multiple-dose clinical pharmacology of the selective orexin-1 receptor antagonist ACT-539313. Prog Neuropsychopharmacol Biol Psychiatry. 2021;108:110166. 33159976 10.1016/j.pnpbp.2020.110166

[19] Kaufmann P Ort M Golor G. First-in-human study with ACT-539313, a novel selec-tive orexin-1 receptor antagonist. Br J Clin Pharmacol. 2020;86:1377–86. 32067262 10.1111/bcp.14251 PMC7319015

[20] Seigneur E de Lecea L. Hypocretin (orexin) replacement therapies. Med Drug Discov. 2020;8:100070. 235 Barson JR. Orexin/hypocretin and dysregulated eating:Promotion of foraging behavior. Brain Res. 2020;1731:145915.

[21] Yamamoto K Okui R Yamatodani A. Activation of orexiner-gic and histaminergic pathway involved in therapeutic effect of histamine H(4) receptor antagonist against cisplatin-induced anorexia in mice. Naunyn Schmiedebergs Arch Pharmacol. 2019;392:925–36. 30919010 10.1007/s00210-019-01646-x

[22] Himmerich H Kan C Katie A. Pharmacological treatment of eating disorders, comorbid mental health problems, malnutrition and physical health consequences. Pharmacol Ther. 2021;217:107667. 32858054 10.1016/j.pharmthera.2020.107667

[23] Chieffi S Carotenuto M Monda V. Orexin system: the key for a healthy life. Front Physiol. 2017;8:357. 28620314 10.3389/fphys.2017.00357 PMC5450021

[24] Karnani MM Apergis-Schoute J Adamantidis A. Activation of central orexin/hypocretin neurons by dietary amino acids. Neuron. 2011;72:616–29. 22099463 10.1016/j.neuron.2011.08.027

[25] Sellayah D Bharaj P Sikder D. Orexin is required for brown adipose tissue development, differentiation, and function. Cell Metab. 2011;14:478–90. 21982708 10.1016/j.cmet.2011.08.010

[26] Kakizaki M Tsuneoka Y Takase K. Differential roles of each orexin receptor signaling in obesity. iScience. 2019;20:1–13. 31546102 10.1016/j.isci.2019.09.003 PMC6817686

[27] Monda V Sessa F Ruberto M. Aerobic exercise and metabolic syndrome: the role of sympathetic activity and the redox system. Diabetes Metab Syndr Obes. 2020;13:2433–42. 32753926 10.2147/DMSO.S257687 PMC7354914

[28] Moretto TL Benfato ID de Carvalho FP. The effects of calorie-matched high-fat diet consumption on spontaneous physical activity and development of obesity. Life Sci. 2017;179:30–6. 28449870 10.1016/j.lfs.2017.04.017

[29] Hao YY Yuan HW Fang PH. Plasma orexin-A level associated with physical activity in obese people. Eat Weight Disord. 2017;22:69–77. 27038345 10.1007/s40519-016-0271-y

[30] Matzeu A Martin-Fardon R. Targeting the orexin system for prescription opioid use disorder. Brain Sci. 2020;10:226. 32290110 10.3390/brainsci10040226 PMC7225970

[31] Mahoney CE Cogswell A Koralnik IJ. The neurobiological basis of narcolepsy. Nat Rev Neurosci. 2019;20:83–93. 30546103 10.1038/s41583-018-0097-x PMC6492289

[32] Kantor S Mochizuki T Lops SN. Orexin gene therapy restores the timing and maintenance of wakefulness in narcoleptic mice. Sleep. 2013;36:1129–38. 23904672 10.5665/sleep.2870 PMC3700709

[33] Black SW Morairty SR Fisher SP. Almorexant promotes sleep and exacerbates cataplexy in a murine model of narcolepsy. Sleep. 2013;36:325–36. 23449602 10.5665/sleep.2442 PMC3571748

[34] Villano I Messina A Valenzano A. Basal forebrain cholinergic system and orexin neurons: effects on attention. Front Behav Neurosci. 2017;11:10. 28197081 10.3389/fnbeh.2017.00010 PMC5281635

[35] Eschenko O Magri C Panzeri S. Noradrenergic neurons of the locus coeruleus are phase locked to cortical up-down states during sleepCereb. Cortex. 2012;22:426–35. 10.1093/cercor/bhr121 21670101

[36] Willie JT Chemelli RM Sinton CM. To eat or to sleep? Orexin in the regulation of feeding and wakefulness. Annu Rev Neurosci. 2001;24:429–58. 11283317 10.1146/annurev.neuro.24.1.429

[37] Willie JT Chemelli RM Sinton CM. Distinct narcolepsy syndromes in orexin receptor-2 and orexin null mice: molecular genetic dissection of Non-REM and REM sleep regulatory processes. Neuron. 2003;38:715–30. 12797957 10.1016/s0896-6273(03)00330-1

[38] Gotter AL Forman MS Harrell CM. Orexin 2 receptor antagonism is sufficient to promote NREM and REM sleep from mouse to man. Sci Rep. 2016;6:27147. 27256922 10.1038/srep27147 PMC4891657

[39] Baimel C Bartlett SE Chiou LC. Orexin/hypocretin role in reward: implications for opioid and other addictions. Br J Pharmacol. 2015;172:334–48. 24641197 10.1111/bph.12639 PMC4292951

[40] Perrey DA Zhang Y. Therapeutics development for addiction: orexin-1 receptor antagonists. Brain Res. 2020;1731:145922. 30148984 10.1016/j.brainres.2018.08.025 PMC6387867

[41] Simmons SJ Gentile TA. Cocaine abuse and midbrain circuits: functional anatomy of hypocretin/orexin transmission and therapeutic prospect. Brain Res. 2020;1731:146164. 30796894 10.1016/j.brainres.2019.02.026 PMC6702109

[42] Campbell EJ Marchant NJ Lawrence AJ. A sleeping giant: suvorexant for the treatment of alcohol use disorder?. Brain Res. 2020;1731:145902. 30081035 10.1016/j.brainres.2018.08.005

[43] Collier AD Min SS Campbell SD. Maternal ethanol consumption before paternal fertilization: stimulation of hypocretin neurogenesis and ethanol intake in zebrafish offspring. Prog Neuropsychopharmacol Biol Psychiatry. 2020;96:109728. 31394141 10.1016/j.pnpbp.2019.109728 PMC6815720

[44] Thannickal TC John J Shan L. Opiates increase the number of hypocretin-producing cells in human and mouse brain and reverse cataplexy in a mouse model of narcolepsy. Sci Transl Med. 2018;10. 10.1126/scitranslmed.aao4953 PMC8235614 29950444

[45] James MH Stopper CM Zimmer BA. Increased number and activity of a lateral subpopulation of hypothalamic orexin/hypocretin neurons underlies the expression of an addicted state in rats. Biol Psychiatry. 2019;85:925–35. 30219208 10.1016/j.biopsych.2018.07.022 PMC7528037

[46] Fragale JE James MH Aston-Jones G. Intermittent self-administration of fentanyl induces a multifaceted addiction state associated with persistent changes in the orexin system. Addict Biol. 2021;26:e12946. 32798290 10.1111/adb.12946 PMC7882007

[47] Mohammadkhani A Fragale Jennifer E Pantazis Caroline B. Orexin-1 receptor signaling in ventral pallidum regulates motivation for the opioid remifentanil. J Neurosci. 2019;39:9831–40. 31641055 10.1523/JNEUROSCI.0255-19.2019 PMC6891064

[48] Plaza-Zabala A Flores A Maldonado R. Hypocretin/orexin signaling in the hypothalamic paraventricular nucleus is essential for the expression of nicotine withdrawal. Biol Psychiatry. 2012;71:214–23. 21831361 10.1016/j.biopsych.2011.06.025

[49] Deats SP Adidharma W Lonstein JS. Attenuated orexinergic signaling underlies depression-like responses induced by daytime light deficiency. Neuroscience. 2014;272:252–60. 24813431 10.1016/j.neuroscience.2014.04.069 PMC4090246

[50] Nollet M Gaillard P Minier F. Activation of orexin neurons in dorsomedial/perifornical hypothalamus and antidepressant reversal in a rodent model of depression. Neuropharmacology. 2011;61:336–46. 21530551 10.1016/j.neuropharm.2011.04.022

[51] Salomon R Ripley B Kennedy JS. Diurnal variation of cerebrospinal fluid hypocretin-1 (Orexin-A) levels in control and depressed subjects. Biol Psychiatry. 2003;54:96–104. 12873798 10.1016/s0006-3223(02)01740-7

[52] Heydendael W Sengupta A Beck S. Optogenetic examination identifies a context-specific role for orex-ins/hypocretins in anxiety-related behavior. Physiol Behav. 2014;130:182–90. 24140988 10.1016/j.physbeh.2013.10.005 PMC4155939

[53] Bonaventure P Dugovic C Shireman B. Evaluation of JNJ-54717793 a novel brain penetrant selective orexin 1 receptor antagonist in two rat models of panic attack provocation. Front Pharmacol. 2017;8:357. 28649201 10.3389/fphar.2017.00357 PMC5465257

[54] Akça OF Uzun N Kilinç İ. Orexin A in adolescents with anxiety disorders. Int J Psychiatry Clin Pract. 2020;24:127–34. 31913740 10.1080/13651501.2019.1711425

